# Whole-genome characterisation of high-risk ST131 and the emerging ST1193 UPEC lineages in Romanian patients with recurrent urinary tract infections

**DOI:** 10.3389/fmicb.2026.1891649

**Published:** 2026-08-12

**Authors:** Laura-Ioana Popa, Sorin Dinu, Daniela Cristea, Andreea Ghiță, Gabriel Ionescu, Mihaela Oprea

**Affiliations:** 1 Genomics, Transcriptomics, and Bioinformatics Platform, Cantacuzino National Military Medical Institute for Research and Development, Bucharest, Romania; 2 Molecular Epidemiology for Communicable Diseases Laboratory, Cantacuzino National Military Medical Institute for Research and Development, Bucharest, Romania; 3 Enteric Bacterial Infections Laboratory - Cantacuzino National Military Medical Institute for Research and Development, Bucharest, Romania; 4 Medical Laboratory - Cantacuzino National Military Medical Institute for Research and Development, Bucharest, Romania; 5 Department II, “Cantacuzino” Microbiology Discipline, Carol Davila University of Medicine and Pharmacy Bucharest, Bucharest, Romania

**Keywords:** antibiotic resistance, ESBL, high-risk clone, recurrent infections, ST1193 *E. coli*, ST131 *E. coli*, UPEC

## Abstract

Recurrent urinary tract infections (UTIs) are frequently associated with persistent uropathogenic *Escherichia coli* (UPEC) lineages that combine antimicrobial resistance with virulence traits, contributing to treatment failure. Whole-genome sequencing was performed on 199 UPEC isolates recovered from patients with recurrent or difficult-to-treat UTIs for whom autovaccine therapy was recommended. Assemblies were analysed using AMRFinderPlus, VFDB, MOB-suite, mobile genetic element detection, serotyping, and clonotype assignment tools, MLST, cgMLST and SNP-based phylogenetic analyses. Recurrent urinary tract infections (UTIs) were associated with multiple pandemic UPEC lineages, including ST131, ST69, ST10 and the emerging ST1193. Genomic analyses identified acquired antimicrobial resistance genes in nearly 80% of isolates, including extended-spectrum *β*-lactamase (ESBL) genes such as *bla*_CTX-M-15_ and *bla*_CTX-M-27_, alongside chromosomal resistance-associated mutations linked to fluoroquinolone resistance. All ST131 and ST1193 isolates fulfilled the molecular UPEC criteria, while all but one isolate was classified as extraintestinal pathogenic *E. coli* (ExPEC). MOB-suite plasmid prediction analysis showed that IncF replicons were the most common and frequently associated with ESBL genes. Almost half of the plasmids were predicted to be mobilizable or conjugative, and one third indicated multireplicon structures. Phylogenetic analyses identified the predominant ST131 O25: H4/CH40-30 and O16: H5/CH40-41 lineages, while most ST1193 isolates belonged to the O75: H5/CH14-64 lineage. Mobile genetic element profiling identified lineage-associated insertion sequence repertoires, class 1 integrons and distinct *bla*_CTX-M_-associated genetic environments. The results indicate that recurrent UTIs in Romania are largely driven by globally disseminated ExPEC clones that combine ESBL production, fluoroquinolone resistance-associated mutations and virulence-associated traits. The detection of closely related isolates recovered in different collection years is consistent with the circulation of successful UPEC lineages within the studied population.

## 1 Introduction

Uropathogenic *Escherichia coli* (UPEC) is the leading cause of one of the most common bacterial infections in humans (Lee et al., 2018; He et al., 2025), being responsible for up to 80–90% of community-acquired urinary tract infections (UTIs; Lee et al., 2018). In some cases, UTIs can cause severe complications such as pyelonephritis, bacteraemia, and septic shock, which may require hospitalisation and long-term treatment (Kot, 2019).

UPEC plays a major role in the global antimicrobial resistance burden, with a rise in prevalence of multidrug-resistant strains, limiting available therapeutic options (Futoma-Kołoch et al., 2025). A major factor is the inadequate use of broad-spectrum antibiotics such as fluoroquinolones, cephalosporins, and aminoglycosides (Sanchez et al., 2016; Kot, 2019). The increase in recurrence rates and antibiotic resistance can be associated with the dissemination of resistance genes through mobile genetic elements (MGEs; Handal et al., 2025).

UPEC strains usually belong to extraintestinal pathogenic *E. coli* (ExPEC), particularly phylogroup B2 (Clermont et al., 2013; Futoma-Kołoch et al., 2025).

Complicated UTIs are frequently associated with multidrug-resistant (MDR) bacteria, particularly ESBL-producing *Escherichia coli* (*E. coli*), the spread of major clones, such as ST131, ST69, ST95, ST10, and ST73, playing an important role in this trend (Riley, 2014; Critchley et al., 2020; Kocsis et al., 2022). The ST131 clone has been previously reported in Romania (Usein et al., 2014; Cristea et al., 2015) and continues to expand in the EU/EEA, carbapenemase-producing *E. coli* ST131 and its SLVs (single locus variants) being detected in multiple countries (Kohlenberg et al., 2024). Within ST131, the O25b: H4 serotype represents the predominant pandemic lineage worldwide (Nicolas-Chanoine et al., 2008, 2014; Mamani et al., 2019; Flament-Simon et al., 2020), while the O16: H5 serotype is recognised as a less common but globally disseminated sublineage associated with the emergence and diversification of this high-risk clone (Nicolas-Chanoine et al., 2014; Mamani et al., 2019; Flament-Simon et al., 2020). More recently, ST1193 has emerged as a globally disseminated fluoroquinolone-resistant ExPEC lineage (Biggel et al., 2021; Pitout et al., 2022). Similar to ST131, it belongs to phylogenetic group B2 and is commonly associated with the O75 serogroup (Pitout and Finn, 2020; Nguyen et al., 2021; Pitout et al., 2022).

The epidemiological success of high-risk UPEC lineages is influenced by a complex interplay of factors extending beyond antimicrobial resistance. In addition to resistance determinants, adaptation of the core genome, virulence-associated traits and the acquisition of MGEs, including plasmids, insertion sequences and integrons, contribute to the long-term maintenance and dissemination of successful clones in both community and healthcare settings (Pitout and Finn, 2020; Decano et al., 2020; Biggel et al., 2022; Zhang et al., 2026).

Plasmids play a crucial role in the evolution of UPEC, particularly IncF-type plasmids, which are commonly associated with ExPEC lineages such as ST131 and carry ESBL genes (Phan et al., 2015; Johnson et al., 2016).

Successful colonisation of the urinary tract depends on various virulence factors, including adhesins, toxins, flagella, and iron-acquisition systems, which support bacterial attachment, invasion, persistence, and evasion of host defences (Terlizzi et al., 2017). The ability of UPEC to persist within protected intracellular and tissue-associated niches further contributes to the chronicity and recurrence of UTIs (De Nisco et al., 2019).

Autovaccines are personalised bacterial vaccines prepared from patient-derived bacterial isolates and have been used in the management of recurrent urinary tract infections. Recent studies have reported reduced recurrence rates and antibiotic consumption following autovaccine administration, supporting their use as an adjunctive strategy in selected patients with recurrent or difficult-to-treat infections (Iftimie et al., 2025; Bonillo-García et al., 2025).

Although Romania is one of the European countries with a very high rate of antibiotic consumption and resistance (OECD/European Observatory on Health Systems and Policies, 2025), surveillance data on such topics are limited (WHO Regional Office for Europe and European Centre for Disease Prevention and Control, 2025). The aim of this study was to identify and characterise UPEC clones causing complicated infections in Romanian patients, using whole-genome sequencing.

## 2 Materials and methods

### 2.1 Microbiological testing

UPEC isolates were detected in urine samples submitted for microbiological analysis at the Cantacuzino National Institute for Medical-Military Research. Samples were mainly collected from patients with recurrent or difficult-to-treat urinary tract infections for whom autovaccine administration had been recommended. Samples were analysed in anonymised form, and no patient identifiers were available. Recurrent urinary tract infection was defined as 2 or more episodes within 6 months or 3 or more episodes within 12 months, according to international clinical guidelines, while difficult-to-treat infections referred to infections associated with persistence despite antimicrobial therapy, MDR isolates, repeated treatment failure, or limited therapeutic options (Anger et al., 2019; Kranz et al., 2024). Bacterial strains were isolated on the non-selective medium Columbia agar supplemented with 5% sheep blood and on the differential medium CLED (Cystine–Lactose–Electrolyte-Deficient) agar. Initial species identification was performed using the Bruker MALDI-TOF MS platform and confirmatory identification was based on conventional biochemical testing. Antimicrobial susceptibility testing was performed using the disk diffusion method in accordance with the current European Committee on Antimicrobial Susceptibility Testing (EUCAST) guidelines and *E. coli* ATCC 25922 strain was used as quality control reference strain. Pure bacterial cultures were preserved and used for genomic DNA extraction. A total of 199 UPEC isolates were included in this study. Most isolates were collected in 2022 (*n* = 133), but isolates were also collected in 2021 (*n* = 27), 2023 (*n* = 10), 2024 (*n* = 22), and 2025 (*n* = 7). The higher number of samples from 2022 is explained by a dedicated institutional project carried out that year, focused on large-scale sequencing using the NovaSeq 6000 platform.

### 2.2 DNA extraction and whole genome sequencing

Genomic DNA was extracted from overnight bacterial cultures using the PureLink™ Genomic DNA Mini Kit (Invitrogen, Thermo Fisher Scientific) according to the manufacturer’s protocol.

DNA concentration was assessed using the Qubit dsDNA High Sensitivity Assay Kit and a Qubit 3 fluorometer (Thermo Fisher Scientific). DNA integrity and fragment size distribution were assessed using the TapeStation System with Genomic DNA ScreenTape and Reagents (Agilent Technologies).

Whole-genome sequencing library preparation was performed using the Nextera XT DNA Library Preparation Kit (Illumina, San Diego, CA, USA) starting from 1 ng of genomic DNA. Dual indexing was done using the IDT for Illumina DNA/RNA UD Index kit (Illumina, San Diego, CA, USA).

Sequencing was done on the Illumina NovaSeq 6,000 platform (Illumina, San Diego, CA, USA) using the NovaSeq 6,000 SP Reagent Kit v1.5, generating paired-end reads of 2 × 150 bp or 2 × 100 bp.

Raw sequencing data were deposited in the European Nucleotide Archive (ENA; Silvester et al., 2018) under BioProject accession PRJEB111143.

### 2.3 Bioinformatics analysis

The quality check on raw data was performed using FastQC^1^ (FastQC, 2018) and *de novo* assemblies was generated with SKESA v2.4.0 (Souvorov et al., 2018), implemented in SeqSphere+ (Ridom GmbH, Münster, Germany; Jünemann et al., 2013).

*E. coli* phylogroups were determined using Clermont Phylotyper according to the Clermont typing scheme—EzClermont (accessed 30 January 2026; Waters et al., 2020). Multilocus sequence typing (MLST) was performed using the Achtman *E. coli* MLST scheme (Wirth et al., 2006) while the core genome MLST (cgMLST) was based on the 2,513 - locus scheme (Jünemann et al., 2013), both implemented within SeqSphere+. Minimum spanning trees based on cgMLST allelic profiles were also generated using SeqSphere+.

The assembled genomes were further analysed using tools embedded within SeqSphere+. AMRFinderPlus (database version 15.11.2023; Feldgarden et al., 2019) was used to identify antimicrobial resistance genes, and the Virulence Factor Database (VFDB—task template version 28.02.2020; Chen et al., 2016) was used to detect virulence-associated genes. Only ARGs passing the threshold identity of ≥ 95% and minimum coverage of ≥ 90% were considered. Gene counts per isolate and classification by antibiotic class were performed in Microsoft Excel using the raw outputs generated by AMRFinderPlus. Virulence-associated genes were selected using the software’s default detection thresholds, extracted from the SeqSphere+ comparison table.

Plasmid detection and characterisation were performed using MOB-suite (MOB-recon version 3.1.4; Robertson and Nash, 2018), also integrated in SeqSphere+, which was applied to the genome assemblies to predict plasmid sequences, determine plasmid mobility types, assign replicon families, and cluster plasmids into related lineages.

In silico serotyping was performed using SeqSphere+ and confirmed using SerotypeFinder (Center for Genomic Epidemiology—CGE; Joensen et al., 2015). Assemblies were used whenever possible, while raw reads were analysed when serotype assignment could not be obtained from draft assemblies. Clonotypes were assigned using CHTyper (CGE) based on *fumC* and *fimH* alleles (Roer et al., 2018).

To further characterise the major lineages ST131 and ST1193, ExPEC and UPEC status were assessed. ExPEC classification was assigned according to the molecular definition proposed by Johnson et al. (2003) based on the presence of at least two of the following markers: *papA*/*papC*, *sfa*/*fo*cDE, *afa*/*draBC*, *kpsM II*, and *iutA*. UPEC status was determined based on the presence of at least three out of the four virulence-associated genes: *chuA*, *fyuA*, *vat*, and *yfcV* (Spurbeck et al., 2012). Virulence genes were identified using the built-in virulence databases available in SeqSphere+ and by additional screening with Abricate implemented on the Galaxy Europe platform (Seemann, 2016) against UPEC/ExPEC-associated virulence factor databases for ST131 and ST1193 isolates. SNP-based phylogenetic analyses of ST131 and ST1193 isolates were performed using Snippy v4.6.0 (Seemann, 2015) on Galaxy Europe. Reads from ST131 and ST1193 isolates were mapped against the complete genome of *Escherichia coli* EC958 (GenBank accession HG941718.1; Forde et al., 2014). The reference genome was downloaded from the NCBI RefSeq database. Core-genome SNP alignments were generated using Snippy-core and phylogenetic trees were reconstructed with FastTree 2 (Price et al., 2010). Tree annotation and visualisation were performed using Interactive Tree of Life (iTOL) v6 (Letunic and Bork, 2024).

Integrons were identified using IntegronFinder implemented in Galaxy Europe (Cury et al., 2016), using default parameters. Mobile genetic elements (MGEs), including insertion sequences (IS), miniature inverted-repeat transposable elements (MITEs), unit transposons and composite transposons, were identified using the Mobile Genetic Elements module available in SeqSphere+ (Jünemann et al., 2013). The genetic environments surrounding *bla*_CTX-M_ genes were investigated using MobileElementFinder (Johansson et al., 2021). Elements spanning contig boundaries and inferred transposons were included in the analysis. Unless otherwise specified, analyses were performed using default software parameters.

Tables and images were generated using Microsoft Excel and Google Docs.

Statistical analyses were performed using non-parametric methods. Differences between two groups were assessed using the Mann–Whitney U test (two-tailed), and comparisons across multiple groups were performed using the Kruskal-Wallis test. Associations between categorical variables were evaluated using Fisher’s exact test. A *p*-value of < 0.05 was considered statistically significant.

## 3 Results

### 3.1 Population structure of the UPEC collection

The study included 199 UPEC isolates, predominantly from female patients (87.9%). Patient age ranged from 8 to 93 years (mean 58.1 years), with similar mean ages observed in male and female patients (60.2 and 57.8 years, respectively).

The isolates were assigned to six phylogroups. B2 was the predominant phylogroup (47%), followed by A (18%), B1 (15%), and D (14%), while C (3.5%) and F (3%) were less frequent.

MLST analysis identified 62 distinct sequence types (STs), of which 39 were represented by a single isolate. ST131 was the most prevalent lineage (18.6%), followed by ST69 (10.1%) and ST1193 (7.5%; Supplementary Figure S1).

ST131 and ST1193 were associated with phylogroup B2, whereas ST69 and ST10 were found in phylogroups D and A, respectively (Supplementary Figure S2).

### 3.2 Antibiotic resistance landscape

#### 3.2.1 Acquired resistance determinants

Among the 199 UPEC isolates included in the study, nearly 80% were predicted to carry at least one acquired ARG, with a maximum of 14 genes detected per isolate. The most prevalent ARGs were associated with resistance to *β*-lactams, identified in 138 isolates (69.3%), followed by resistance to aminoglycosides in 117 isolates (58.8%), resistance to tetracyclines in 104 isolates (52.3%), resistance to trimethoprim in 105 isolates (52.7%), and resistance to sulphonamides in 99 isolates (49.2%). Macrolide resistance genes were observed in 54 isolates (27.1%), genes conferring resistance to quinolones were identified in 31 isolates (13.6%), while phenicol, lincosamide, and fosfomycin resistance determinants were less common, being detected in 27 (12.1%), 1 (0.5%) and 1 (0.5%) isolate, respectively. Multiple resistance genes associated with the same antibiotic class were frequently observed, particularly for aminoglycosides (up to 5). The complete list of detected resistance genes is provided in Supplementary Table S1.

Among the 174 β-lactam resistance gene occurrences identified across 138 isolates, *bla*_TEM_ was the most prevalent family, with *bla*_TEM-1_ being the most frequently identified gene (*n* = 82). ESBL-associated CTX-M variants were also common, particularly *bla*_CTX-M-15_ (*n* = 29), closely followed by *bla*_CTX-M-27_ (*n* = 22), whereas other alleles such as *bla*_CTX-M-3_, *bla*_CTX-M-1_, *bla*_CTX-M-32_, *bla*_CTX-M-55_, and *bla*_CTX-M-14_ were rarely detected (*n* = 1–5). OXA-type β-lactamase genes were identified at a lower frequency (*n* = 20), while carbapenem resistance determinants were rare, with only a single isolate carrying *bla*_OXA-181_ (*n* = 1; ST4981). *bla*_CARB-2_ gene, encoding a narrow-spectrum carbenicillanase, was detected in one isolate (Supplementary Table S1).

A total of 109 tetracycline resistance genes (in 104 isolates) were found across the collection. The predominant alleles were *tet*(A) (*n* = 67) and *tet*(B) (*n* = 40), which together accounted for 98.2% of all determinants. In contrast, *tet*(M) was rarely identified (*n* = 2).

Among the 105 *dfrA* genes encoding trimethoprim resistance, the most prevalent allele was *dfrA17* (*n* = 60), accounting for more than half of the total determinants. Other detected variants included *dfrA5* (*n* = 13), *dfrA14* (*n* = 11), *dfrA1* (*n* = 10), and *dfrA12* (*n* = 7). The alleles *dfrA7* (*n* = 3) and *dfrA16* (*n* = 1) were observed sporadically.

Within the aminoglycoside class, a total of 296 ARGs were identified. Most isolates harboured multiple determinants, ranging from 1 to 5 genes per isolate. Genes encoding aminoglycoside-modifying enzymes were dominated by phosphotransferases (*aph*), most prevalent being *aph(6)-Id* (*n* = 75), *aph(3″)-Ib* (*n* = 74), and *aph(3′)-Ia* (*n* = 24). Among adenylyltransferases (*aadA*), the most prevalent were *aadA5* (*n* = 54), *aadA1* (*n* = 22), and *aadA2* (*n* = 14), while acetyltransferase genes (aac) were less frequent, with *aac(3)-IId* being the most common variant.

A total of 137 sulphonamide resistance genes were identified, with *sul2* (*n* = 72) and *sul1* (*n* = 57) as the predominant genes, while *sul3* was less frequently identified (*n* = 8). Of the 99 positive isolates, 61 carried a single gene while 38 carried two determinants.

ARGs conferring resistance to other antibiotic classes were detected at a lower frequency.

#### 3.2.2 Chromosomal mutation-associated resistance

**Figure 1:**
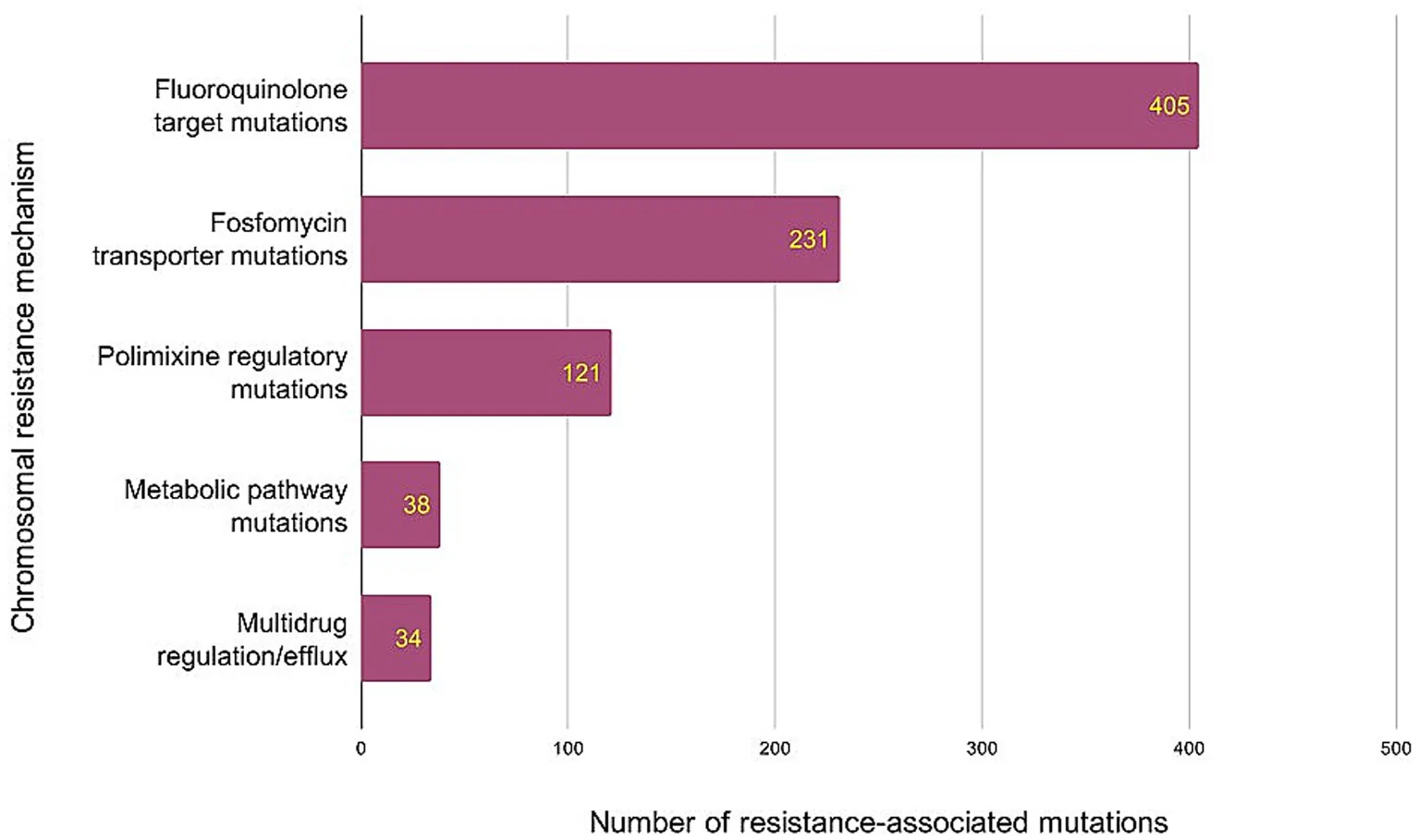
Distribution of chromosomal resistance-associated mutations by mechanism. Bars represent the total number of mutations identified for each resistance mechanism.

A total of 25 chromosomal mutations associated with antimicrobial resistance were detected. Fosfomycin resistance-associated mutations were present in 181 isolates (91%). Mutations in *glpT* gene were the most prevalent, found in 164 isolates (82.4%), whereas *uhpT* mutations were identified in 67 isolates (33.6%), most carrying a single mutation. A total of 121 isolates (60.8%) harboured mutations in genes previously associated with colistin resistance. We identified two substitutions in the *pmrB* gene: *pmrB* E123D, present in 84 isolates (42.2%), and *pmrB* Y358N, detected in 37 isolates (18.6%), each isolate carrying a single colistin-associated substitution, which has been described in both colistin-resistant and susceptible isolates. Fluoroquinolone resistance showed the most complex mutational profile, with 405 resistance-associated mutations found across 111 positive isolates (55.8%). These substitutions were most frequently observed in *gyrA*, *parC*, and *parE*, with multiple mutations frequently co-occurring within individual isolates, the most prevalent being *gyrA* S83L (*n* = 103, 51.7%), *gyrA* D87N (*n* = 87, 43.7%), and *parC* S80I (*n* = 91, 45.7%). Mutations in regulatory and metabolic genes were less frequently detected, *ptsI* V25I being present in 38 isolates (19.1%). Similarly, multidrug/efflux-associated substitutions were identified in 33 isolates (16.6%) carrying the *marR* S3N mutation and a single isolate (0.5%) carrying *soxS* A12S (Figure 1; Supplementary Table S1).

To investigate the distribution of chromosomal point mutations involved in antibiotic resistance, isolates with more than 5 resistance-associated mutations were grouped by identical profiles (Supplementary Figure S3). The largest number of mutations was observed in 28 isolates belonging to ST131 and a SLV, which exhibited 9 substitutions affecting polymyxin-, fosfomycin-, and fluoroquinolone-associated genes.

### 3.3 Virulence gene repertoire

Across all isolates, 37 virulence-associated genes were detected. Isolates belonging to ExPEC-associated phylogroups (B2, D, and F) showed higher virulence gene counts, while non-ExPEC/commensal-associated phylogroups (A, B1, and C) displayed lower counts. This difference was statistically significant (Mann–Whitney U test, *p* < 0.001).

The adhesion profile was dominated by nearly universal determinants (*fim* - 96.98%, *fdeC*-95.98%, *yag/ecp*-97.49%), while *pap* (57.29%) and *sfa* (51.76%) were present in over half of the isolates. Iron acquisition systems were well represented. Enterobactin-associated genes were ubiquitous (*ent*-100%, *fep*-100%, *fes*-99.5%), while hem uptake (chu-63.82%) and additional siderophore systems (*iuc*-59.8%, *iro*-37.19%) were present in a consistent subset. Protective/survival determinants were frequent (*kps*-59.3%) or ubiquitous (*ompA*-100%), while secreted toxins/effectors were less common (*sat-*33.67% and *hly*- 24.12%, *cnf1*-14.57%). One ST10 isolate carried Shiga toxin-encoding genes (*stxA2d*/*stxB2c*), representing an atypical virulence profile within the collection (Supplementary Table S1).

### 3.4 Predicted plasmid clusters (MOB-suite and ARG association)

Plasmid analysis identified 769 predicted plasmid sequences ranging from 0.99 kb to 270 kb (average size 36.3 kb). Approximately half were non-mobilizable (52%), while 25% were mobilizable and 23% conjugative. Replicon typing showed a predominance of IncF-family plasmids, with IncFIB, IncFIA, IncFIC, and IncFII being the most frequent types. Notably, one-third of plasmids carried multiple replicons, most commonly within the IncF family (Supplementary Table S2).

We identified 207 plasmid clusters based on MOB-suite classification, including 38 groups that could not be assigned to previously described lineages. While several clusters were broadly distributed across the collection, many were small, cryptic plasmids. The most abundant cluster, AG884 (*n* = 53), consists of small (mean size 1.5 kb) non-mobilizable elements, with no replicon type or ARGs associated, and is rarely associated with virulence genes such as iron-acquisition or colicin systems.

In contrast, in the cluster AA176 (*n* = 37) there are large plasmids (mean size: 128 kb), most of them with mobilisation potential. These plasmids were predominantly predicted as single-replicon, with Col156 as the dominant type, and were further divided into several secondary putative clusters, of which two lineages (AH856 and AH858) were predominant. The AH856 lineage displayed variable resistance content, carrying 0–7 ARGs per plasmid, typically including *sul1*, *bla*_TEM-1_, aminoglycoside and tetracycline resistance genes. By contrast, AH858 showed a more conserved MDR content, with 6–10 ARGs per plasmid and frequent co-occurrence of sul1, *bla*_TEM-1_, *tet*(A), and *dfrA17* (Supplementary Figure S4).

Another notable cluster, AA171 (*n* = 29), contained large plasmids (47–150 kb) belonging to a single replicon family (Col(MG828)), most of which were predicted to be mobilizable. The predominant subcluster, AH824, displayed a conserved ESBL-associated backbone, with most plasmids carrying *bla*_CTX-M_ variants, particularly *bla*_CTX-M-27_. Some of them also harboured additional MDR determinants, with some plasmids encoding more than 10 ARGs conferring resistance to multiple antibiotic classes, while another smaller subcluster (AH828) harboured the *bla*_OXA-1_ gene (Supplementary Figure S5).

Additional lower-frequency putative clusters added to the diversity of predicted plasmids. Cluster AA179 contained a small set of IncF-type plasmids displaying mixed mobility profiles and carrying up to seven ARGs, including *bla*_CTX-M-15_ and *bla*_CTX-M-55_, which were detected only twice across the entire collection.

### 3.5 Integrated resistance-virulence results

Phylogroup B2 exhibited the highest average number of virulence determinants, along with a high resistance gene number, while phylogroups D and F also showed high virulence despite variable resistance levels (Supplementary Figure S6). In contrast, commensal-associated phylogroups (A, B1, C) displayed lower numbers of virulence genes despite a similar or higher average number of antibiotic resistance determinants. Despite the significant difference in virulence gene content, there is no clear association between the number of ARG and phylogroups (Mann–Whitney U test, *p* > 0.05).

Phylogroup B2 exhibited a variable content of antibiotic resistance and virulence genes across STs. However, isolates belonging to the same ST tend to display similar resistance profiles across antibiotic classes and comparable virulence gene repertoires. Notably, ESBL genes were significantly associated with phylogroup B2 (Fisher test, *p* < 0.01).

### 3.6 Major UPEC clones

**Figure 2:**
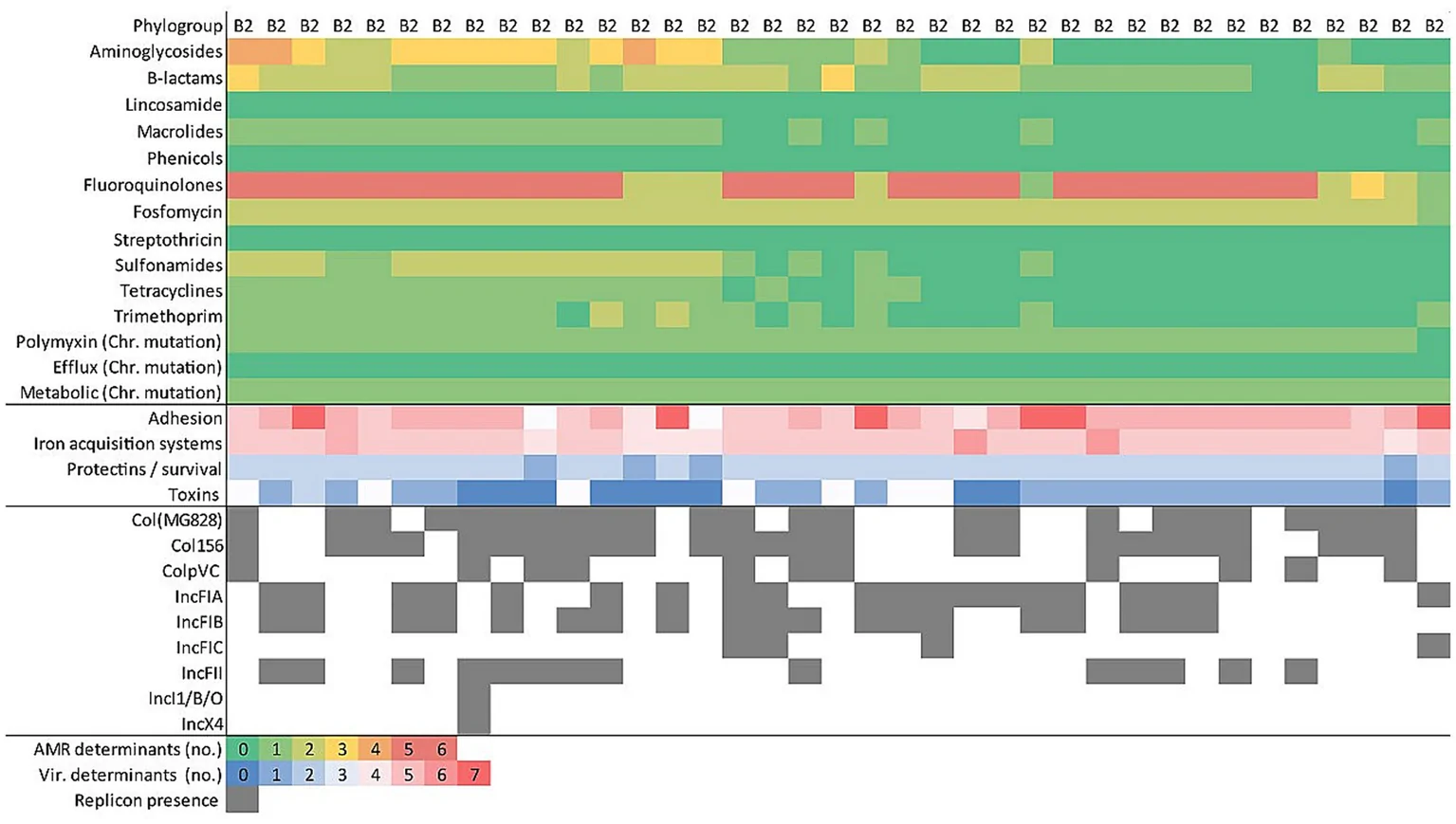
Heatmap showing resistance determinants, virulence genes, and plasmid replicons in ST131 *E. coli* isolates. Rows represent functional categories and columns represent individual isolates. Colours indicate feature counts per isolate using the scales shown in the figure, while grey indicates the presence of plasmid replicons. Resistance determinants include both acquired antimicrobial resistance genes and chromosomal mutations, some of which have been previously associated with resistance but do not necessarily confer phenotypic resistance.

ST131 isolates showed a high number of resistance determinants to *β*-lactams, including ESBL genes, and also to fluoroquinolones and fosfomycin across nearly all strains (Figure 2). In silico serotyping revealed that most ST131 isolates belonged to serotype O25: H4 (27/37, 73.0%), followed by O16: H5 (8/37, 21.6%), while two isolates could not be assigned an O antigen and were classified as OND: H4. Clonotype analysis identified CH40-30 as the dominant lineage (28/37, 75.7%), whereas all O16: H5 isolates belonged to CH40-41 (8/37, 21.6%). A single O25: H4 isolate was assigned to CH40-99. All ST131 isolates fulfilled the UPEC criteria, while 36/37 (97.3%) were classified as ExPEC (Supplementary Table S3). Furthermore, mutations in polymyxin regulatory genes (*pmrB*) were detected in a subset of isolates. ESBL genes were significantly associated with ST131 isolates (Fisher test, *p* < 0.01). Approximately half of the isolates also carried genes and substitutions associated with resistance to aminoglycosides, macrolides, sulphonamides, tetracyclines, and trimethoprim. The total number of resistance determinants varied considerably between isolates, ranging from 6 to 22 determinants per isolate. Virulence gene content was also variable, ranging from 8 to 20 determinants, although most isolates carried 13–16 virulence-associated genes.

ST131 isolates often carried multireplicon IncF plasmids (IncFIA/IncFIB +/− IncFII; Figure 2), which were frequently linked to ESBL genes, especially *bla*_CTX-M_ variants (notably CTX-M-27 and CTX-M-15).

**Figure 3:**
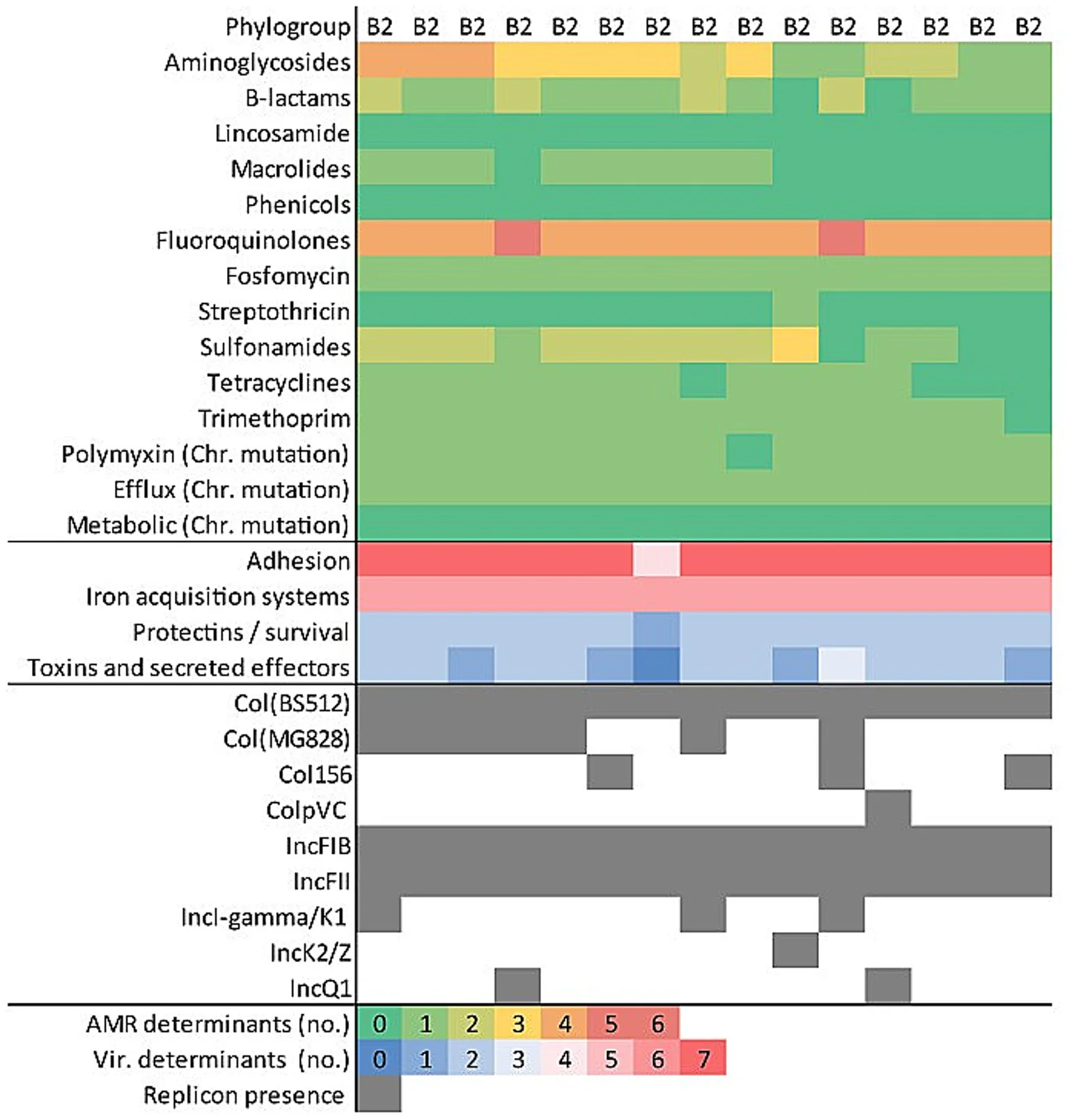
Heatmap showing resistance determinants, virulence genes, and plasmid replicons in ST1193 *E. coli* isolates. Rows represent functional categories and columns represent individual isolates. Colours indicate feature counts per isolate using the scales shown in the figure, while grey indicates the presence of plasmid replicons. Resistance determinants include both acquired antimicrobial resistance genes and chromosomal mutations, some of which have been previously associated with resistance but do not necessarily confer phenotypic resistance.

ST1193 also exhibited a high number of resistance determinants (9–17 per isolate), with consistent presence of resistance determinants associated with resistance to fluoroquinolones, aminoglycosides, and fosfomycin and frequent resistance to *β*-lactams, sulphonamides, and other antibiotic classes (Figure 3). Serotype analysis showed a strong predominance of O75: H5 (13/15, 86.7%), while O18: H5 and O18ac: H5 were each identified in a single isolate. All ST1193 isolates belonged to clonotype CH14-64 and fulfilled both ExPEC and UPEC classification criteria. Detailed serotype, clonotype, ExPEC/UPEC status, the presence of QRDR, β-lactamase content and integron distribution for ST131 and ST1193 isolates are presented in Supplementary Table S4. Virulence gene content ranged from 10 to 16.

ST1193 strains showed a diverse plasmid profile with IncFIB and IncFII replicons commonly detected and frequently co-occurring with additional replicon types, including IncI-gamma/K, IncK2/Z, and IncQ1. ESBL determinants (e.g., *bla*_CTX-M-3_) were less commonly associated with IncF backbones. Moreover, ST1193 plasmids frequently carried virulence-associated determinants, such as the aerobactin operon (*iucABC*) and *senB*.

Phenotypic antimicrobial susceptibility data were available for a subset of ST131 and ST1193 isolates (Supplementary Table S4). Full concordance was observed between the presence of *bla*_CTX-M_ genes and ceftazidime resistance. Among norfloxacin-resistant isolates, all but one carried the classical QRDR profile investigated in this study. The remaining resistant isolate lacked the classical combination but harboured an alternative combination of fluoroquinolone resistance-associated mutations (*gyrA* S83L, *parE* I529L and *parC* E84K,). Similarly, the combination of *dfrA* and *sul* genes explained the trimethoprim-sulfamethoxazole resistance phenotype in the majority of isolates. Three exceptions were observed: one resistant isolate carried a *dfrA* gene without a sul determinant, one lacked both *dfrA* and *sul* genes, and one carried only a partial *dfrA17* sequence (86% coverage), which was not initially identified as a complete resistance determinant. All tested isolates remained susceptible to fosfomycin despite the presence of previously reported chromosomal variants, including *glpT* E448K, *uhpT* E350Q, and *ptsI* V25I.

All ST10 isolates carried up to 14 ARGs per isolate, although some isolates lacked detectable resistance genes (Supplementary Figure S7). The highest number of resistance determinants was observed for fluoroquinolones, aminoglycosides and beta-lactams. Virulence gene counts ranged from 8 to 20.

ST69, the next most prevalent ST, displayed a range of ARGs between 1 and 17, with consistent resistance to fosfomycin and variable resistance to aminoglycosides, *β*-lactams, macrolides, fluoroquinolones, sulphonamides, tetracyclines, and trimethoprim. Virulence gene content ranged from 8 to 17 genes, mainly associated with adhesion and iron acquisition (Supplementary Figure S7).

ST73, another globally disseminated UPEC clone, exhibited the lowest number of ARGs (*n* = 2–8). Despite the low antibiotic resistance burden, strains displayed a high virulence gene content ranging from 17 to 20, involved in adhesion, iron acquisition systems, toxin production, and secreted effectors (Supplementary Figure S7).

The number of antimicrobial resistance determinants varied significantly across these STs (Kruskal-Wallis test, *p* < 0.001), with higher values observed in ST131 and ST1193 compared to the other lineages. In contrast, virulence gene content showed an opposite pattern, with the highest values observed in ST73, followed by ST69 (Kruskal-Wallis test, *p* < 0.001).

### 3.7 Phylogenetic analysis of major clones

**Figure 4:**
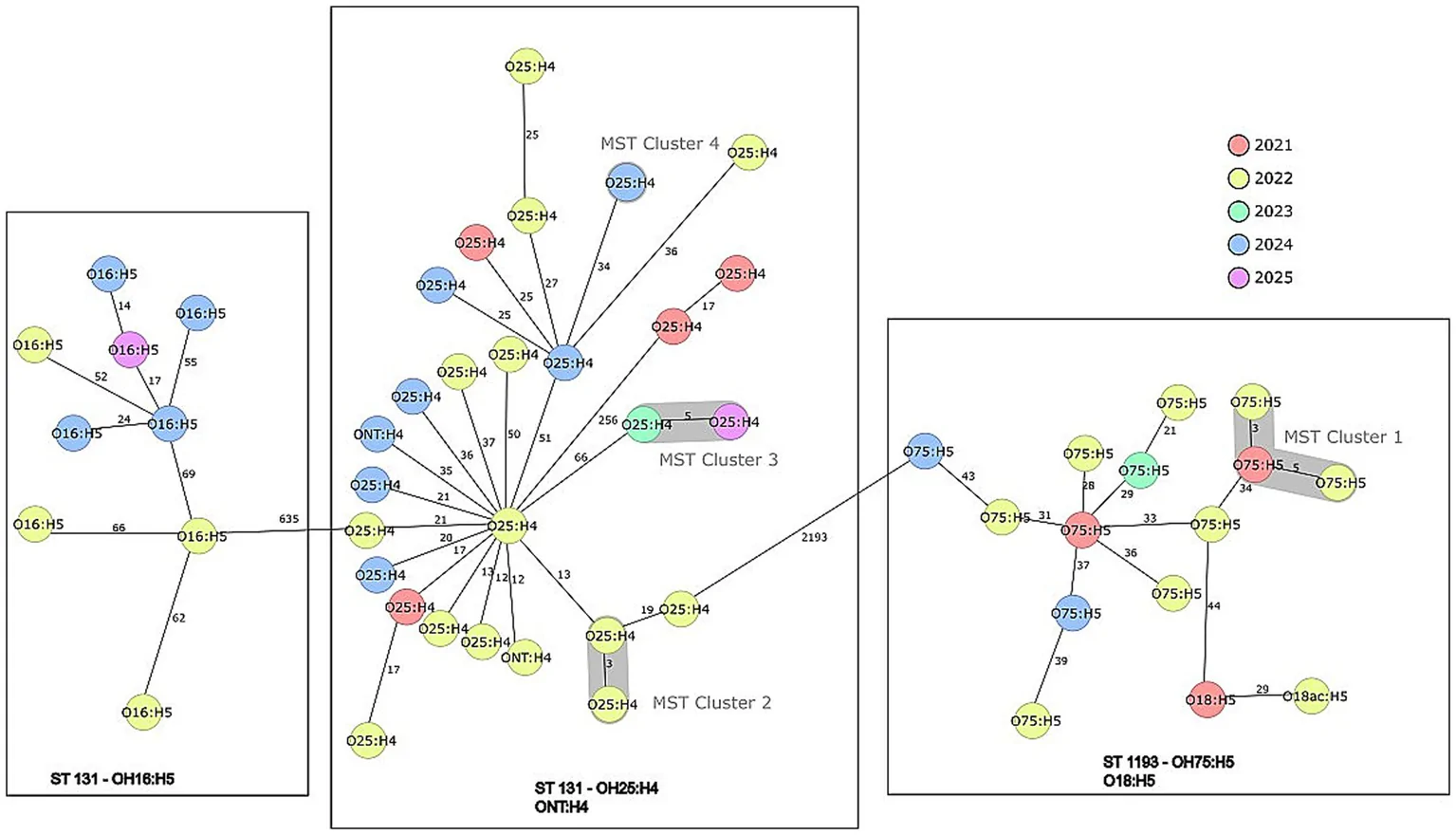
cgMLST-based minimum spanning tree of ST131 isolates. Nodes represent isolates, and distances correspond to allelic differences. Clusters were defined using a threshold of ≤10 allelic differences.

cgMLST-based minimum spanning tree analysis revealed a clear separation between ST131 and ST1193 isolates (Figure 4). Within ST131, isolates were further separated into two major groups corresponding to the O25: H4 and O16: H5 serotypes, whereas ST1193 isolates formed a distinct group dominated by the O75: H5 serotype.

Three closely related ST131 isolate pairs with only 0–5 allele differences were identified. Notably, one pair included isolates recovered in 2023 and 2025 despite no known epidemiological link. Among ST1193 isolates, a small cluster of three closely related strains (3–5 allele differences) was observed, including isolates collected in both 2021 and 2022.

**Figure 5:**
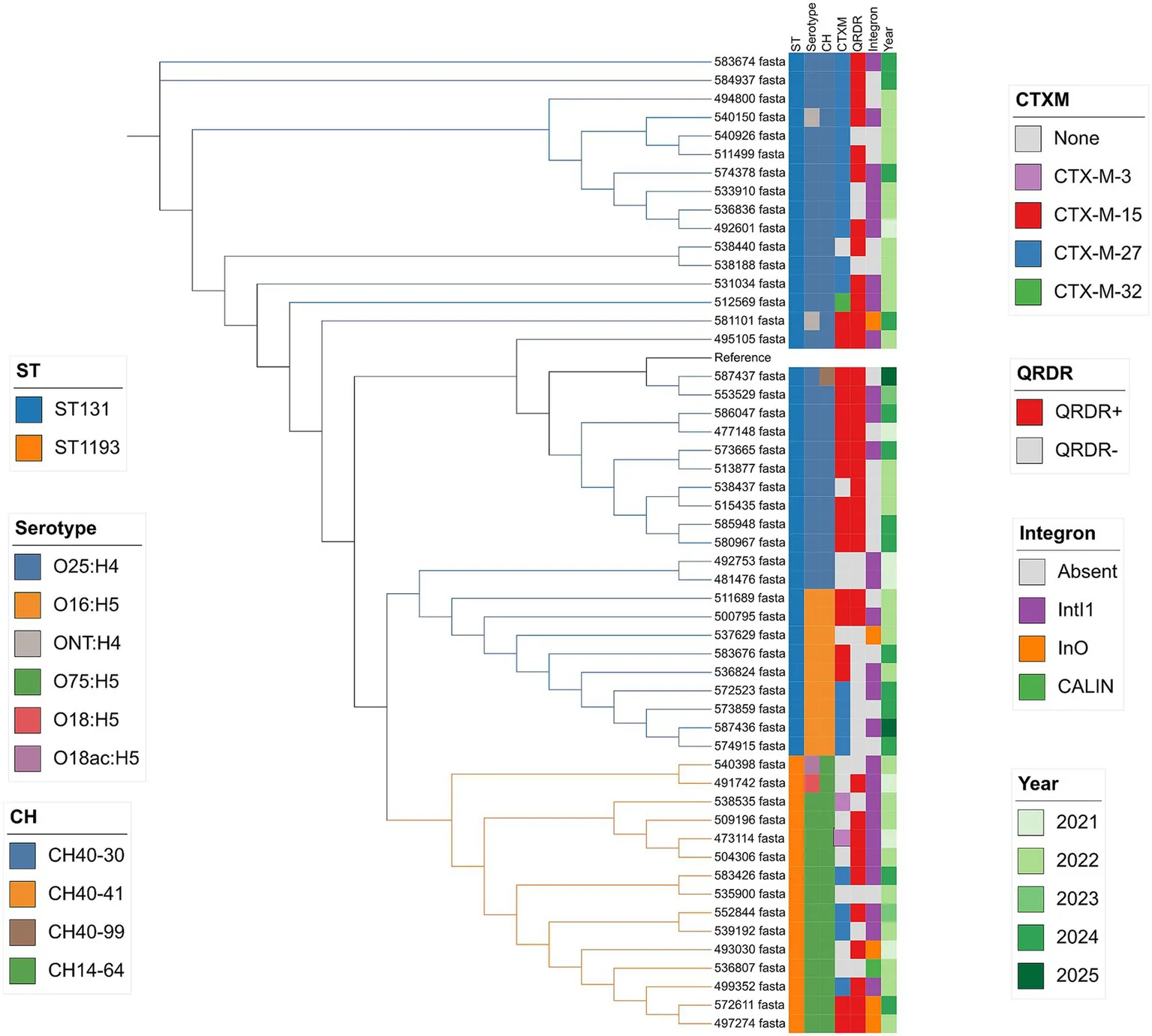
SNP-based phylogenetic trees of ST131 and ST1193 isolates with associated genomic characteristics. Colored annotation tracks represent serotype, clonotype (CH), *bla*CTX-M allele, QRDR profile, integron status, and year of isolation. The color key for each category is shown alongside the phylogeny. The reference strain *Escherichia coli* EC958 is indicated on the tree.

SNP-based phylogenetic analyses of ST131 and ST1193 are shown in Figure 5. The ST131 phylogeny showed a clear separation between isolates belonging to the O25: H4 and O16: H5 serotypes, corresponding to the predominant CH40-30 and CH40-41 clonotypes. Isolates carrying *bla*_CTX-M-15_ and *bla*_CTX-M-27_ were identified in multiple branches of the tree. Within the O25: H4 lineage, isolates carrying *bla*_CTX-M-15_ and *bla*_CTX-M-27_ tended to group in different parts of the phylogeny, although neither allele was restricted to a single lineage. ST1193 showed a more homogeneous structure, with most isolates belonging to the O75: H5/CH14-64 lineage. The classical QRDR profile and ESBL-associated *bla*_CTX-M_ genes including the less frequent *bla*_CTX-M-3_ allele identified in two isolates, were detected in several isolates distributed throughout the tree.

### 3.8 Integrons, mobile genetic elements and blaCTX-M genetic contexts

Integron-associated structures were identified in both major lineages (Supplementary Table S3). Among ST131 isolates, class 1 integrons (IntI) were detected in 18/37 isolates, while two additional isolates carried In0 structures lacking gene cassettes. Among ST1193 isolates, class 1 integrons were more frequent, being detected in 10/15 isolates. Three ST1193 isolates carried In0 structures and one isolate contained a CALIN (cluster of attC sites lacking an integrase gene) element.

Mobile genetic element analysis revealed distinct profiles between the two lineages. ST131 isolates were characterised by the frequent presence of IS30, ISEc1, ISEc38, ISEc53 and ISKpn8, whereas ST1193 isolates showed a more conserved profile dominated by IS100, IS609, ISEc30, ISEc31, ISEc40 and ISEc42. MITEEc1 was identified in all isolates belonging to both lineages. Transposons were less frequently detected and included Tn5403 in a subset of ST131 isolates.

Distinct genetic environments were observed among ESBL-associated *bla*_CTX-M_ genes. In ST131, *bla*_CTX-M-15_ was commonly associated with ISEc9/ISEcp1-like elements, whereas *bla*_CTX-M-27_ was typically identified in association with IS903-like sequences and truncated ISEc9 elements. The single *bla*_CTX-M-32_-positive isolate carried an ISEc9-*bla*_CTX-M-32_-ISKpn26 arrangement. In ST1193, *bla*_CTX-M-15_ was associated with ISEc9, while *bla*_CTX-M-27_ was repeatedly detected in an IS102-*bla*_CTX-M-27_-truncated ISEc9 configuration. Detailed MGE profiles and *bla*_CTX-M_-associated genetic environments are presented in Supplementary Table S3.

## 4 Discussion

This study provides a genomic overview of UPEC isolates associated with recurrent urinary tract infections. Globally disseminated clones such as high-risk ST131 (Forde et al., 2019), alongside the emerging ST1193 lineage (Pitout et al., 2022; Zeng et al., 2025), belonging to phylogroup B2 lineages, dominated the UPEC population. Other clinically relevant lineages, including ST69, ST10, and ST73, were also common, while ST95 was detected sporadically (Riley, 2014; Garcia et al., 2025). To the best of our knowledge, this is the first whole-genome sequencing-based investigation of human UPEC isolates from Romania, providing key insights into strains predominantly associated with recurrent UTIs.

Previous Romanian studies have reported a predominance of phylogroup B2 among UPEC isolates, particularly among strains from outpatients, patients with neurogenic bladder, and vaginal *E. coli* strains carrying multiple ExPEC-associated virulence genes (Mladin et al., 2009; Usein et al., 2011). ESBL-associated resistance was more frequently reported among non-B2 lineages, especially phylogroup A (Porumbel et al., 2018; Cristea et al., 2019). In contrast, the present study identified a predominance of B2 clones carrying both resistance and virulence determinants, including ESBL genes. Previous Romanian collections, where MDR rates were approximately 35% and ESBL prevalence around 9% in community-acquired UPEC (Cristea et al., 2019) or 19% in paediatric UTIs (Păcurar et al., 2025), displayed lower counts compared to our research, where predicted genomic MDR rates were approximately 70%, and ESBL genes (namely *bla*_CTX-M_) were detected in approximately 31% of isolates. This is in accordance with other Romanian collections that reported a high proportion of CTX-M genes among clinical isolates (Usein et al., 2014) and also consistent with global trends, as the incidence of extended-spectrum *β*-lactamase-producing *E. coli* is on the rise, CTX-M-15 being the most commonly detected (Critchley et al., 2020). Phenotypic susceptibility testing performed for a subset of ST131 and ST1193 isolates showed complete concordance between the presence of *bla*_CTX-M_ genes and resistance to ceftazidime.

The predominance of the ST131 clone observed in our study is consistent with earlier reports from Romania, which documented its emergence among clinical *E. coli* isolates from invasive infections (Usein et al., 2014; Cristea et al., 2015).

Serotype and clonotype analyses further supported the presence of globally disseminated high-risk sublineages. Most ST131 isolates belonged to the pandemic O25: H4/CH40-30 lineage, whereas a smaller subset was assigned to O16: H5/CH40-41, a recognised ST131 clade A-associated sublineage (Nicolas-Chanoine et al., 2014; Mamani et al., 2019; Flament-Simon et al., 2020). ST1193 showed a more homogeneous structure, with all isolates belonging to clonotype CH14-64 and most assigned to serotype O75: H5, consistent with the global expansion of a highly conserved fluoroquinolone-resistant lineage (Tchesnokova et al., 2019; Pitout et al., 2022).

Previous studies have shown that mobile genetic elements, such as integrons, contribute to the dissemination of resistance determinants among UPEC strains carrying gene cassettes encoding resistance to trimethoprim, aminoglycosides, *β*-lactams, and chloramphenicol (Oprea et al., 2020). These structures were predominantly detected in phylogroups A and B1, suggesting that earlier resistance dissemination was mostly driven by non-B2 lineages.

The *bla*_CARB-2_ has been previously reported in *Klebsiella pneumoniae* on a conjugative plasmid (Zhu et al., 2018) and in *Salmonella Typhimurium* associated with integrons (Monte et al., 2020). In our dataset, *bla*_CARB-2_ was also predicted to be located on a plasmid and was detected in a phylogroup A isolate.

High rates of fluoroquinolone resistance have been reported in Romanian clinical settings (Chibelean et al., 2020), consistent with the frequent detection of fluoroquinolone resistance determinants (especially point mutations) in the present genomic analysis. While carbapenem resistance remained rare, with only one isolate carrying *bla*_OXA-181_, fosfomycin resistance genes were common in our collection. Several β-lactamase genes, particularly *bla*_TEM_ and *bla*_OXA_, were identified only at the family level, therefore, their specific functional classification (e.g., ESBL or carbapenemase) could not be determined and they were considered as general β-lactam resistance determinants.

Mutations associated with fluoroquinolone resistance were frequently observed in our isolates, especially the quinolone resistance-associated genes *gyrA* and *parC*. The most prevalent substitutions were *gyrA* S83L, *gyrA* D87N, and *parC* S80I, which contribute to high-level ciprofloxacin resistance, especially when carrying multiple mutations within the same isolate (Redgrave et al., 2014). Phenotypic susceptibility testing available for a subset of ST131 and ST1193 isolates showed an almost complete concordance between fluoroquinolone resistance and the quinolone resistance-determining region (QRDR; Johnning et al., 2015). All but one norfloxacin-resistant isolate carried the classical combination of gyrA S83L, gyrA D87N, and parC S80I (Johnning et al., 2015). The remaining resistant isolate harboured an alternative mutation profile (gyrA S83L, parC E84K, and parE I529L), suggesting that combinations outside the classical QRDR pattern may also contribute to the resistant phenotype.

Substitutions in the *pmrB* identified in this study have previously been reported in association with colistin-resistant isolates (Hao et al., 2022), however more recent genomic analyses demonstrated that variants such as E123D and Y358N are not associated with phenotypic colistin resistance but instead correlate with phylogenetic background, being frequently detected in susceptible isolates (Butters et al., 2024).

High point mutation resistance-associated profiles were associated with specific STs rather than being randomly distributed across the collection. The most prevalent profile, consisting of nine mutations affecting polymyxin regulatory genes (*pmrB*), fosfomycin, and fluoroquinolone-associated resistance genes, was shared by 28 isolates belonging to ST131, with another isolate from the same ST131 clonal complex (ST18758), followed by 14 ST1193 samples sharing the same pattern. This pattern indicates clonal dissemination of a conserved mutational background mainly driven by clonal expansion of high-risk UPEC lineages rather than independent mutation events.

A similar observation was made for fosfomycin-associated substitutions. Although variants in *glpT*, *uhpT*, and *ptsI* were frequently identified, all phenotypically tested isolates remained susceptible to fosfomycin. Fosfomycin resistance is most commonly associated with impaired drug uptake caused by alterations in the GlpT and UhpT transport systems or in their regulatory pathways, including ptsI and cyaA (Falagas et al., 2016), however, the phenotypic impact depends on the nature of the genetic alteration rather than simply on the presence of a sequence variant. Experimental studies have shown that complete loss-of-function mutations or transporter disruption are associated with markedly increased fosfomycin MICs (Ballestero-Téllez et al., 2017), whereas amino acid substitutions have been reported in phenotypically susceptible *E. coli* isolates (Hurwitz et al., 2024).

Longitudinal data from a Northern California community demonstrated that a limited number of pandemic UPEC lineages persist for long periods and influence local resistance trends, with six major STs accounting for over 60% of UTIs across 17 years. While ST131 was rarely encountered (3–5%), it contributed to the emergence of fluoroquinolone resistance (Yamaji et al., 2018).

In our dataset, substitutions associated with fluoroquinolone resistance were also frequently detected, particularly in isolates belonging to ST131 and ST1193, consistent with their role in the accumulation of resistance mechanisms (Nicolas-Chanoine et al., 2014; Pitout and DeVinney, 2017; Tchesnokova et al., 2019).

In addition to antimicrobial resistance, isolates belonging to phylogroup B2 and, overall, the ExPEC group, had a higher number of virulence genes than commensal-associated phylogroups. All ST1193 isolates and all but one ST131 isolate fulfilled the molecular criteria for ExPEC classification, while all isolates from both lineages met the UPEC definition. The high prevalence of adhesion determinants and iron acquisition systems likely improves the ability of these to colonise the urinary tract and persist within the host, supporting their role as successful ExPEC lineages (Flores-Mireles et al., 2015; Sora et al., 2021).

Interestingly, virulence gene content was highest in ST73 compared to ST131 and ST1193. Since ST73 carried a low antibiotic resistance burden, this may suggest a different pattern of adaptation in these lineages.

Hybrid Shiga toxin (*stx*)-producing *Escherichia coli* (STEC) and UPEC strains were previously described (Bielaszewska et al., 2014). We detected one ST 10 isolate recovered from a urinary tract infection carrying *stxA2d*/*stxB2c*. Besides STEC-associated virulence factors, this isolate also carried genes involved in adhesion and iron acquisition, and immune evasion, but lacks classical UPEC-defining markers such as *pap*, *sfa* or *kpsM*. Overall, this suggests that this STEC isolate may also be capable of causing infection in an extraintestinal niche (Gati et al., 2021).

The plasmid analysis showed considerable diversity in predicted plasmid clusters, approximately half showing mobilisation potential, including both mobilizable and conjugative elements. This suggests that horizontal gene transfer likely plays an important role in the dissemination of resistance determinants within the studied UPEC population (Partridge et al., 2018).

Many ST131 isolates carried IncF-type plasmids. Similar associations between ST131 and F-type plasmids have been previously described (Phan et al., 2015; Johnson et al., 2016). Among our isolates, these plasmids were frequently associated with ESBL genes, most often *bla*_CTX-M_ variants such as CTX-M-15 and CTX-M-27, an association previously described in analyses of ST131 plasmids (Mnif et al., 2013; Kondratyeva et al., 2020; Martínez-Álvarez et al., 2025). This indicates that IncF plasmids are likely involved in the dissemination of the ESBL determinants within this lineage. Many of the predicted plasmids were classified as multireplicon elements, most commonly involving combinations of IncF replicons. These plasmids were previously reported in ExPEC/UPEC strains and have been linked to the dissemination of ESBL determinants within high-risk lineages such as ST131 (Martínez-Álvarez et al., 2025).

Plasmid reconstruction in this study relied on predictions generated with MOB-suite from draft genome assemblies (Robertson and Nash, 2018). Previous studies have shown that this tool performs well for reconstructing ARG-associated plasmids in *E. coli* genomes (Paganini et al., 2021). Although short-read assemblies provide reliable information on plasmid diversity, replicon composition, and mobility potential, they may fragment plasmid sequences and occasionally yield incomplete or hybrid plasmid reconstructions (Robertson and Nash, 2018).

Despite the diversity of insertion sequences detected across the collection, the genetic environments associated with *bla*_CTX-M_ genes were remarkably conserved. *Bla*_CTX-M-15_ was consistently associated with ISEcp1-like (ISEc9) elements in both ST131 and ST1193, whereas *bla*_CTX-M-27_ was linked to IS903-like structures in ST131 and to an IS102-associated platform in ST1193. ISEcp1 has been recognised as one of the principal elements involved in the mobilisation and expression of *bla*_CTX-M_ genes, including *bla*_CTX-M-15_ (Poirel et al., 2005; Partridge et al., 2018). Similar IS903-associated genetic environments have been described for the globally disseminated ST131 C1-M27 lineage, where *bla*_CTX-M-27_ is typically embedded within conserved ΔISEcp1–*bla*_CTX-M-27_–IS903D structures (Matsumura et al., 2016; Birgy et al., 2017). The repeated detection of these arrangements across unrelated isolates and multiple collection years suggests the successful dissemination and maintenance of stable ESBL-associated mobilisation platforms within the Romanian UPEC population.

The phylogenetic analysis revealed distinct population structures within the two major lineages. Within ST131, SNP-based analysis resolved the O25: H4/CH40-30 and O16: H5/CH40-41 sublineages, consistent with the recognised population structure of this high-risk clone (Nicolas-Chanoine et al., 2014; Matsumura et al., 2017; Flament-Simon et al., 2020). Several closely related ST131 and ST1193 isolates were recovered in different years, suggesting the persistence of successful lineages within the studied population. Most ST1193 isolates belonged to the O75: H5/CH14-64 lineage, consistent with previous reports describing this clone as a highly conserved emerging ExPEC lineage (Tchesnokova et al., 2019; Pitout et al., 2022).

The SNP phylogeny further highlighted the internal diversity of the O25: H4 ST131 lineage. Although isolates carrying *bla*_CTX-M-15_ and *bla*_CTX-M-27_ were identified throughout the phylogeny, a partial segregation of the two ESBL determinants was observed within the O25: H4 background. This pattern is consistent with previous studies showing that the global success of ST131 has been driven by the expansion of multiple sublineages that differ in their associated ESBL content. In particular, *bla*_CTX-M-15_ has been strongly associated with the H30Rx/C2 lineage, whereas *bla*_CTX-M-27_ has been linked to the more recently emerged C1-M27 lineage, both belonging to the O25: H4 serotype background (Matsumura et al., 2016; Birgy et al., 2017; Flament-Simon et al., 2020). Although formal subclade assignment was not performed in the present study, the observed phylogenetic distribution of *bla*_CTX-M_ variants suggests that multiple successful O25: H4-associated lineages may be circulating within the Romanian UPEC population.

The predominance of ST131, ST69, and ST1193 in the present collection is consistent with previous studies showing that pandemic UPEC clones can persist within the intestinal microbiota and act as reservoirs for recurrent infections caused by the same strain (Forde et al., 2019). The globally disseminated lineages belonging to the B2 phylogroup, particularly ST131 and ST1193, combine multidrug resistance, virulence, and mobile genetic elements, facilitating long-term colonisation, spread, persistence and recurrent infections (Pitout, 2012; Pitout et al., 2022). Given the high burden of antimicrobial resistance and the presence of high-risk clones, our results highlight the need for improved regional surveillance and public health programmes in Romania.

This study has several limitations. The isolates were collected mostly in 2022, with limited samples from other years, and may not fully represent the diversity of UPEC circulating at the national level. In addition, phenotypic antimicrobial susceptibility data were available only for a subset of ST131 and ST1193 isolates, limiting genotype–phenotype comparisons across the entire collection. Although predicted genomic MDR rates were high, not all resistance-associated variants translated into phenotypic resistance. This was particularly evident for fosfomycin, where all tested isolates remained susceptible despite carrying substitutions in *glpT*, *uhpT*, and *ptsI*. Although virulence-associated genes were identified by whole-genome sequencing, their expression and contribution to pathogenicity were not experimentally validated. Moreover, plasmid reconstruction relied on predictions from short-read sequencing, which may lead to incomplete results.

Because patient-level longitudinal data were unavailable and isolates were analysed anonymously, persistence at the individual patient level could not be assessed. Therefore, references to persistence in this study refer to the continued detection of related clonal lineages within the collection over multiple years rather than persistence of infection within individual patients.

## 5 Conclusion

This study provides a comprehensive genomic characterisation of UPEC isolates associated with recurrent and difficult-to-treat urinary tract infections in Romanian patients. Multidrug-resistant B2-associated lineages, particularly ST131 and the emerging ST1193 clone, were frequently identified and were characterised by distinct serotype-clonotype combinations, extensive antimicrobial resistance determinants, and enriched virulence-associated profiles. SNP-based phylogenetic analyses identified the predominant ST131 O25: H4/CH40-30 and O16: H5/CH40-41 lineages, while most ST1193 isolates belonged to the O75: H5/CH14-64 lineage. The widespread occurrence of ESBL-associated *bla*_CTX-M_ genes, fluoroquinolone resistance-associated mutations, and conserved mobile genetic element-associated resistance platforms highlights the successful adaptation and continued circulation of these lineages within the studied population. Together, these findings emphasise the importance of genomic surveillance for monitoring the evolution and dissemination of clinically relevant ExPEC lineages and antimicrobial resistance determinants in Romania.

## Data Availability

The datasets presented in this study can be found in online repositories. The names of the repository/repositories and accession number(s) can be found in the article/Supplementary material.

## Footnotes

1
https://www.bioinformatics.babraham.ac.uk/projects/fastqc

## Supplementary material

SUPPLEMENTARY FIGURE S1 Distribution of major sequence types (ST131, ST69, ST1193, ST10, and ST73) across the study period (2021-2025). Stacked bar plot showing the annual number of isolates for each sequence type.

SUPPLEMENTARY FIGURE S2 Distribution of major sequence types (ST131, ST69, ST1193, ST10, and ST73) across phylogenetic groups. Stacked bar plot showing the number of isolates per phylogroup. Less frequent sequence types were grouped as “Others”.

SUPPLEMENTARY FIGURE S3 Average number of resistance determinants (including both acquired resistance genes and chromosomal mutations) and virulence factors per isolate across phylogenetic groups. Bars represent the mean number of detected features per isolate within each phylogroup.

SUPPLEMENTARY FIGURE S4 Distribution of chromosomal resistance-associated mutation profiles among isolates carrying more than five mutations. Isolates were grouped based on identical mutational patterns across polymyxin-, fosfomycin-, and fluoroquinolone-associated genes.

SUPPLEMENTARY FIGURE S5 Structure and resistance gene content of plasmid cluster AA176 and associated lineages. Plasmids are grouped by MOB-suite classification, showing ARG content and predicted mobility.

SUPPLEMENTARY FIGURE S6 Structure and resistance gene content of plasmid cluster AA171. Highlighting ESBL-associated *bla*_CTX-M_ variants and multidrug resistance profiles.

SUPPLEMENTARY FIGURE S7 Resistance and virulence profiles of the rest of the isolates. Heatmap representation showing variability in antimicrobial resistance determinants and virulence gene content. Colours indicate feature counts per isolate using the scales shown in the figure. Resistance determinants include both acquired antimicrobial resistance genes and chromosomal mutations, some of which have been previously associated with resistance but do not necessarily confer phenotypic resistance.

SUPPLEMENTARY TABLE S1 Complete list of detected antimicrobial resistance genes, chromosomal mutations, and virulence factors across all isolates.

SUPPLEMENTARY TABLE S2 Predicted plasmid sequences, replicon types, mobility classification, and associated ARGs.

SUPPLEMENTARY TABLE S3 Additional characteristics of ST131 and ST1193 isolates.

SUPPLEMENTARY TABLE S4 Phenotypic antimicrobial susceptibility profiles and associated resistance determinants in ST131 and ST1193 isolates.

## References

[ref1] Anger J. Lee U. Ackerman A. L. Chou R. Chughtai B. Clemens J. Q. . (2019). Recurrent uncomplicated urinary tract infections in women: AUA/CUA/SUFU guideline. J. Urol. 202, 282–289. doi: 10.1097/JU.0000000000000296, 31042112

[ref2] Ballestero-Téllez M. Docobo-Pérez F. Rodríguez-Martínez J. M. Conejo M. C. Ramos-Guelfo M. S. Blázquez J. . (2017). Role of *uhpT*, *glpT*, *cyaA*, and *ptsI* genes in fosfomycin resistance in *Escherichia coli*. J. Antimicrob. Chemother. 72, 1303–1311. doi: 10.1093/jac/dkw574 28093485

[ref3] Bielaszewska M. Schiller R. Lammers L. Bauwens A. Fruth A. Middendorf B. . (2014). Heteropathogenic virulence and phylogeny reveal phased pathogenic metamorphosis in *Escherichia coli* O2:H6. EMBO Mol. Med. 6, 347–357. doi: 10.1002/emmm.201303133, 24413188 PMC3958309

[ref4] Biggel M. Hoehn S. Schmitt K. Frei A. Jans C. Stephan R. (2021). Complete genome sequence of *Escherichia coli* sequence type 1193 isolate AVS0096 recovered from river water in Switzerland. Microbiol. Resour. Announc. 10:e00607-21. doi: 10.1128/MRA.00607-21 34351235 PMC8340865

[ref5] Biggel M. Moons P. Nguyen M. N. Goossens H. Van Puyvelde S. (2022). Convergence of virulence and antimicrobial resistance in increasingly prevalent *Escherichia coli* ST131 papGII+ sublineages. Commun. Biol. 5:752. doi: 10.1038/s42003-022-03660-x, 35902767 PMC9334617

[ref6] Birgy A. Bidet P. Levy C. Sobral E. Cohen R. Bonacorsi S. (2017). CTX-M-27-producing *Escherichia coli* of sequence type 131 and clade C1-M27, France. Emerg. Infect. Dis. 23:885. doi: 10.3201/eid2305.161865, 28418829 PMC5403054

[ref7] Bonillo-García M. Á. Ordaz-Jurado D. G. Ortiz-Salvador J. Colet-Guitert J. O. Morán-Pascual E. Martínez-Cuenca E. . (2025). Autovaccine immunoprophylaxis in patients with neurogenic bladder experiencing recurrent urinary tract infections. Front. Immunol. 16:1626422. doi: 10.3389/fimmu.2025.1626422, 40821772 PMC12354533

[ref8] Butters A. Jovel J. Gow S. Liljebjelke K. Waldner C. Checkley S. L. (2024). PmrB Y358N, E123D amino acid substitutions are not associated with colistin resistance but with phylogeny in *Escherichia coli*. Microbiol. Spectrum 12, e00532–e00524. doi: 10.1128/spectrum.00532-24, 39162501 PMC11451601

[ref9] Chen L. Zheng D. Liu B. Yang J. Jin Q. (2016). VFDB 2016: hierarchical and refined dataset for big data analysis—10 years on. Nucleic Acids Res. 44, D694–D697. doi: 10.1093/nar/gkv1239, 26578559 PMC4702877

[ref10] Chibelean C. B. Petca R. C. Mareș C. Popescu R. I. Enikő B. Mehedințu C. . (2020). A clinical perspective on the antimicrobial resistance spectrum of uropathogens in a Romanian male population. Microorganisms 8:848. doi: 10.3390/microorganisms8060848, 32516902 PMC7357063

[ref11] Clermont O. Christenson J. K. Denamur E. Gordon D. M. (2013). The Clermont *Escherichia coli* phylo-typing method revisited: improvement of specificity and detection of new phylo-groups. Environ. Microbiol. Rep. 5, 58–65. doi: 10.1111/1758-2229.12019, 23757131

[ref12] Cristea V. C. Gheorghe I. Czobor Barbu I. Popa L. I. Ispas B. Grigore G. A. . (2019). Snapshot of phylogenetic groups, virulence, and resistance markers in *Escherichia coli* uropathogenic strains isolated from outpatients with urinary tract infections in Bucharest, Romania. Biomed. Res. Int. 2019:5712371. doi: 10.1155/2019/5712371 31236408 PMC6545812

[ref13] Cristea V. C. Oprea M. Neacşu G. Gîlcă R. Popa M. I. Usein C. R. (2015). Mechanisms of resistance to ciprofloxacin and genetic diversity of *Escherichia coli* strains originating from urine cultures performed for Romanian adults. Roum. Arch. Microbiol. Immunol. 74, 73–78. 27328520

[ref14] Critchley I. A. Cotroneo N. Pucci M. J. Jain A. Mendes R. E. (2020). Resistance among urinary tract pathogens collected in Europe during 2018. J. Glob. Antimicrob. Resist. 23, 439–444. doi: 10.1016/j.jgar.2020.10.020, 33212286

[ref9001] Cury J. Jové T. Touchon M. Néron B. Rocha E. P. C. (2016). Identification and analysis of integrons and cassette arrays in bacterial genomes. Nucleic Acids Res. 44, 4539–4550. doi: 10.1093/nar/gkw319, 27130947 PMC4889954

[ref15] De Nisco N. J. Neugent M. Mull J. Chen L. Kuprasertkul A. de Souza Santos M. . (2019). Direct detection of tissue-resident bacteria and chronic inflammation in the bladder wall of postmenopausal women with recurrent urinary tract infection. J. Mol. Biol. 431, 4368–4379. doi: 10.1016/j.jmb.2019.04.008, 31002774 PMC6801050

[ref16] Decano A. G. Tran N. Al-Foori H. . (2020). Plasmids shape the diverse accessory resistomes of *Escherichia coli* ST131. Access Microbiol. 3:000179. doi: 10.1099/acmi.0.000179, 33997610 PMC8115979

[ref17] Falagas M. E. Vouloumanou E. K. Samonis G. Vardakas K. Z. (2016). Fosfomycin. Clin. Microbiol. Rev. 29, 321–347. doi: 10.1128/CMR.00068-15, 26960938 PMC4786888

[ref18] FastQC. (2018). FastQC: a quality control tool for high throughput sequence data (Version 0.11.7). Available online at: https://www.bioinformatics.babraham.ac.uk/projects/fastqc/ (Accessed April 16, 2025).

[ref19] Feldgarden M. Brover V. Haft D. H. Prasad A. B. Slotta D. J. Tolstoy I. . (2019). Validating the AMRFinder tool and resistance gene database by using antimicrobial resistance genotype-phenotype correlations in a collection of isolates. Antimicrob. Agents Chemother. 63, e00483–e00419. doi: 10.1128/AAC.00483-19, 31427293 PMC6811410

[ref20] Flament-Simon S. C. García V. Duprilot M. Mayer N. Alonso M. P. García-Meniño I. . (2020). High prevalence of ST131 subclades C2-H30Rx and C1-M27 among extended-spectrum β-lactamase-producing *Escherichia coli* causing human extraintestinal infections in patients from two hospitals of Spain and France during 2015. Front. Cell. Infect. Microbiol. 10:125. doi: 10.3389/fcimb.2020.00125, 32266173 PMC7105571

[ref21] Flores-Mireles A. L. Walker J. N. Caparon M. Hultgren S. J. (2015). Urinary tract infections: epidemiology, mechanisms of infection and treatment options. Nat. Rev. Microbiol. 13, 269–284. doi: 10.1038/nrmicro3432, 25853778 PMC4457377

[ref22] Forde B. M. Ben Zakour N. L. Stanton-Cook M. Phan M. D. Totsika M. Peters K. M. . (2014). The complete genome sequence of *Escherichia coli* EC958: a high-quality reference sequence for the globally disseminated multidrug-resistant *E. coli* O25b:H4-ST131 clone. PLoS One 9:e104400. doi: 10.1371/journal.pone.0104400, 25126841 PMC4134206

[ref23] Forde B. M. Roberts L. W. Phan M. D. Peters K. M. Fleming B. A. Russell C. W. . (2019). Population dynamics of an *Escherichia coli* ST131 lineage during recurrent urinary tract infection. Nat. Commun. 10:3643. doi: 10.1038/s41467-019-11571-5, 31409795 PMC6692316

[ref24] Futoma-Kołoch B. Sarowska J. Abd El-Salam M. Miñana-Galbis D. Drabová B. Wiśniewska P. . (2025). Current insights into antibiotic resistance in uropathogenic *Escherichia coli and* interventions using selected bioactive phytochemicals. Antibiotics 14:1242. doi: 10.3390/antibiotics14121242, 41463744 PMC12729374

[ref25] Garcia B. B. Paiva G. E. Silva M. U. B. (2025). High-risk *Escherichia coli* global clones ST10 and ST155 in wild raptors admitted to a rehabilitation center. Vet. Res. Commun. 49:241. doi: 10.1007/s11259-025-10811-y, 40591063

[ref26] Gati N. S. Temme I. J. Middendorf-Bauchart B. Kehl A. Dobrindt U. Mellmann A. (2021). Comparative phenotypic characterization of hybrid Shiga toxin-producing/uropathogenic *Escherichia coli*, canonical uropathogenic and Shiga toxin-producing *Escherichia coli*. Int. J. Med. Microbiol. 311:151533. doi: 10.1016/j.ijmm.2021.151533, 34425494

[ref27] Handal N. Kaspersen H. Mo S. S. Cabanel N. Jørgensen S. B. Fortineau N. . (2025). A comparative study of the molecular characteristics of human uropathogenic *Escherichia coli* collected from two hospitals in Norway and France in 2019. J. Antimicrob. Chemother. 80, 1707–1715. doi: 10.1093/jac/dkaf130, 40296582 PMC12129582

[ref28] Hao J. Zeng Z. Xiao X. Ding Y. Deng J. Wei Y. . (2022). Genomic and phenotypic characterization of a colistin-resistant *Escherichia coli* isolate co-harboring *bla*NDM-5, *bla*OXA-1, and *bla*CTX-M-55 isolated from urine. Infect. Drug Resist. 15, 1329–1343. doi: 10.2147/IDR.S355010, 35378893 PMC8976530

[ref29] He Y. Zhao J. Wang L. Han C. Yan R. Zhu P. . (2025). Epidemiological trends and predictions of urinary tract infections in the global burden of disease study 2021. Sci. Rep. 15:4702. doi: 10.1038/s41598-025-89240-5, 39922870 PMC11807111

[ref30] Hurwitz J. S. Newton-Foot M. Nel van Zyl K. Nel P. (2024). Fosfomycin susceptibility testing and resistance mechanisms in Enterobacterales in South Africa. Afr. J. Lab. Med 13:2252. doi: 10.4102/ajlm.v13i1.2252 38629086 PMC11019110

[ref31] Iftimie S. Ladero-Palacio P. López-Azcona A. F. Pujol-Galarza L. Pont-Salvadó A. Gabaldó-Barrios X. . (2025). Evaluating the use of Uromune® autovaccine in recurrent urinary tract infections: a pilot unicenter retrospective study in Reus. Spain. BMC Infect. Dis. 25:117. doi: 10.1186/s12879-025-10524-2, 39856603 PMC11762519

[ref32] Joensen K. G. Tetzschner A. M. Iguchi A. Aarestrup F. M. Scheutz F. (2015). Rapid and easy in silico serotyping of Escherichia coli using whole genome sequencing (WGS) data. J.Clin.Microbiol. 53, 2410–2426. doi: 10.1128/JCM.00008-15 25972421 PMC4508402

[ref9002] Johansson M. H. K. Bortolaia V. Tansirichaiya S. Aarestrup F. M. Roberts A. P. Petersen T. N. (2021). Detection of mobile genetic elements associated with antibiotic resistance in Salmonella enterica using a newly developed web tool: MobileElementFinder. J. Antimicrob. Chemother. 76, 101–109. doi: 10.1093/jac/dkaa390 33009809 PMC7729385

[ref33] Johnning A. Kristiansson E. Angelin M. Marathe N. Shouche Y. S. Johansson A. . (2015). Quinolone resistance mutations in the faecal microbiota of Swedish travellers to India. BMC Microbiol. 15:235. doi: 10.1186/s12866-015-0574-6, 26498929 PMC4619388

[ref34] Johnson T. J. Danzeisen J. L. Youmans B. Case K. Llop K. Munoz-Aguayo J. . (2016). Separate F-type plasmids have shaped the evolution of the H30 subclone of *Escherichia coli* sequence type 131. mSphere 1e00121–16. doi: 10.1128/mSphere.00121-16, 27390780 PMC4933990

[ref35] Johnson J. R. Murray A. C. Gajewski A. Sullivan M. Snippes P. Kuskowski M. A. . (2003). Isolation and molecular characterization of nalidixic acid-resistant extraintestinal pathogenic *Escherichia coli* from retail chicken products. Antimicrob. Agents Chemother. 47, 2161–2168. doi: 10.1128/AAC.47.7.2161-2168.2003 12821463 PMC161843

[ref36] Jünemann S. Sedlazeck F. J. Prior K. Albersmeier A. John U. Kalinowski J. . (2013). Updating benchtop sequencing performance comparison. Nat. Biotechnol. 31, 294–296. doi: 10.1038/nbt.2522, 23563421

[ref37] Kocsis B. Gulyás D. Szabó D. (2022). Emergence and dissemination of extraintestinal pathogenic high-risk international clones of *Escherichia coli*. Life 12:2077. doi: 10.3390/life12122077, 36556442 PMC9780897

[ref38] Kohlenberg A. Svartström O. Apfalter P. Hartl R. Bogaerts P. Huang T.-D. . (2024). Emergence of *Escherichia coli* ST131 carrying carbapenemase genes, European Union/European economic area, august 2012 to may 2024. Euro Surveill. 29:2400727. doi: 10.2807/1560-7917.ES.2024.29.47.2400727, 39574387 PMC11583312

[ref39] Kondratyeva K. Salmon-Divon M. Navon-Venezia S. (2020). Meta-analysis of pandemic *Escherichia coli* ST131 plasmidome proves restricted plasmid-clade associations. Sci. Rep. 10:36. doi: 10.1038/s41598-019-56763-7, 31913346 PMC6949217

[ref40] Kot B. (2019). Antibiotic resistance among uropathogenic *Escherichia coli*. Pol. J. Microbiol. 68, 403–415. doi: 10.33073/pjm-2019-048, 31880885 PMC7260639

[ref41] Kranz J. Bartoletti R. Bruyère F. Cai T. Geerlings S. Köves B. . (2024). European association of urology guidelines on urological infections: summary of the 2024 guidelines. Eur. Urol. 86, 27–41. doi: 10.1016/j.eururo.2024.03.035, 38714379

[ref42] Lee D. S. Lee S.-J. Choe H.-S. (2018). Community-acquired urinary tract infection by *Escherichia coli* in the era of antibiotic resistance. Biomed. Res. Int. 2018:7656752. doi: 10.1155/2018/7656752, 30356438 PMC6178185

[ref43] Letunic I. Bork P. (2024). Interactive tree of life (iTOL) v6: recent updates to the phylogenetic tree display and annotation tool. Nucleic Acids Res. 52, W78–W82. doi: 10.1093/nar/gkae268, 38613393 PMC11223838

[ref9003] Mamani R. Flament-Simon S. C. García V. Mora A. Alonso M. P. López C. . (2019). Sequence types, clonotypes, serotypes, and virotypes of extended-spectrum β-lactamase-producing Escherichia coli causing bacteraemia in a Spanish hospital over a 12-year period (2000 to 2011). Front. Microbiol. 10:1530. doi: 10.3389/fmicb.2019.01530, 31379759 PMC6646471

[ref44] Martínez-Álvarez S. A. Asencio-Egea M. Á. Huertas-Vaquero M. Cardona-Cabrera T. Zarazaga M. Höfle U. . (2025). Genomic epidemiology of ESBL- and carbapenemase-producing Enterobacterales in a Spanish hospital: exploring the clinical-environmental interface. Microorganisms 13:1854. doi: 10.3390/microorganisms13081854, 40871359 PMC12388269

[ref45] Matsumura Y. Pitout J. D. Gomi R. Matsuda T. Noguchi T. Yamamoto M. . (2016). Global *Escherichia coli* sequence type 131 clade with blaCTX-M-27 gene. Emerg. Infect. Dis. 22, 1900–1907. doi: 10.3201/eid2211.160519, 27767006 PMC5088012

[ref46] Matsumura Y. Pitout J. D. D. Peirano G. DeVinney R. Noguchi T. Yamamoto M. . (2017). Rapid identification of different *Escherichia coli* sequence type 131 clades. Antimicrob. Agents Chemother. 61, e00179–e00117. doi: 10.1128/AAC.00179-17, 28584160 PMC5527616

[ref47] Mladin C. Usein C. R. Chifiriuc M. C. Andi-Palade M. Slavu C. L. Negut M. . (2009). Genetic analysis of virulence and pathogenicity features of uropathogenic *Escherichia coli* isolated from patients with neurogenic bladder. Rom. Biotechnol. Lett. 14, 4900–4905.

[ref48] Mnif B. Harhour H. Jdidi J. Mahjoubi F. Genel N. Arlet G. . (2013). Molecular epidemiology of extended-spectrum beta-lactamase-producing *Escherichia coli* in Tunisia and characterization of their virulence factors and plasmid addiction systems. BMC Microbiol. 13:147. doi: 10.1186/1471-2180-13-147, 23800277 PMC3701463

[ref49] Monte D. F. M. Sellera F. P. Lopes R. Keelara S. Landgraf M. Greene S. . (2020). Class 1 integron-borne cassettes harboring blaCARB-2 gene in multidrug-resistant and virulent *Salmonella Typhimurium* ST19 strains recovered from clinical human stool samples, United States. PLoS One 15:e0240978. doi: 10.1371/journal.pone.0240978, 33125394 PMC7598458

[ref50] Nguyen Q. Nguyen T. T. N. Pham P. Chau V. Nguyen L. P. H. Nguyen T. D. . (2021). Genomic insights into the circulation of pandemic fluoroquinolone-resistant extra-intestinal pathogenic *Escherichia coli* ST1193 in Vietnam. Microb. Genom. 7:000733. doi: 10.1099/mgen.0.000733, 34904942 PMC8767341

[ref51] Nicolas-Chanoine M. H. Bertrand X. Madec J. Y. (2014). *Escherichia coli* ST131, an intriguing clonal group. Clin. Microbiol. Rev. 27, 543–574. doi: 10.1128/CMR.00125-13, 24982321 PMC4135899

[ref52] Nicolas-Chanoine M. H. Blanco J. Leflon-Guibout V. Demarty R. Alonso M. P. Caniça M. M. . (2008). Intercontinental emergence of *Escherichia coli* clone O25:H4-ST131 producing CTX-M-15. J. Antimicrob. Chemother. 61, 273–281. doi: 10.1093/jac/dkm464, 18077311

[ref53] OECD/European Observatory on Health Systems and Policies (2025). Country Health Profile 2025: Romania. Paris/Brussels: OECD Publishing/European Observatory on Health Systems and Policies.

[ref54] Oprea M. Militaru M. C. Ciontea A. S. Cristea D. Cristea V. Usein C. R. (2020). Characterization of antibiotic resistance integrons harbored by Romanian *Escherichia coli* uropathogenic strains. Rev. Rom. Med. Lab. 28, 331–340. doi: 10.2478/rrlm-2020-0023

[ref55] Păcurar D. Dinulescu A. Totu A. V. Dijmărescu I. Pavelescu M. L. (2025). *Escherichia coli* urinary tract infections from a Romanian pediatric hospital: antimicrobial resistance trends, ESBL prevalence, and empirical treatment implications. Antibiotics 14:855. doi: 10.3390/antibiotics14090855, 41009834 PMC12466793

[ref56] Paganini J. A. Plantinga N. L. Arredondo-Alonso S. Willems R. J. L. Schürch A. C. (2021). Recovering *Escherichia coli* plasmids in the absence of long-read sequencing data. Microorganisms 9:1613. doi: 10.3390/microorganisms9081613, 34442692 PMC8400445

[ref57] Partridge S. R. Kwong S. M. Firth N. Jensen S. O. (2018). Mobile genetic elements associated with antimicrobial resistance. Clin. Microbiol. Rev. 31, e00088–e00017. doi: 10.1128/cmr.00088-17, 30068738 PMC6148190

[ref58] Phan M. D. Forde B. M. Peters K. M. Sarkar S. Hancock S. Stanton-Cook M. . (2015). Molecular characterization of a multidrug resistance IncF plasmid from the globally disseminated *Escherichia coli* ST131 clone. PLoS One 10:e0122369. doi: 10.1371/journal.pone.0122369, 25875675 PMC4398462

[ref59] Pitout J. D. D. (2012). Extraintestinal pathogenic *Escherichia coli*: a combination of virulence with antibiotic resistance. Front. Microbiol. 3:9. doi: 10.3389/fmicb.2012.00009, 22294983 PMC3261549

[ref60] Pitout J. D. DeVinney R. (2017). *Escherichia coli* ST131: a multidrug-resistant clone primed for global domination. F1000Res. 6:195. doi: 10.12688/f1000research.10609.1, 28344773 PMC5333602

[ref61] Pitout J. D. D. Finn T. J. (2020). The evolutionary puzzle of *Escherichia coli* ST131. Infect. Genet. Evol. 81:104265. doi: 10.1016/j.meegid.2020.104265, 32112974

[ref62] Pitout J. D. D. Peirano G. Chen L. DeVinney R. Matsumura Y. (2022). *Escherichia coli* ST1193: following in the footsteps of *E. coli* ST131. Antimicrob. Agents Chemother. 66:e0051122. doi: 10.1128/aac.00511-22, 35658504 PMC9295538

[ref63] Poirel L. Lartigue M. F. Decousser J. W. Nordmann P. (2005). ISEcp1B-mediated transposition of *bla*_CTX-M_ in *Escherichia coli*. Antimicrob. Agents Chemother. 49, 447–450. doi: 10.1128/AAC.49.1.447-450.2005, 15616333 PMC538921

[ref64] Porumbel I. Chelariu M. Gheorghe I. Curutiu C. Popa M. Delcaru C. . (2018). Genotypic characterization of virulence and resistance markers in ESBL positive *Escherichia coli* strains isolated from ambulatory urinary tract infections. Rom. Biotechnol. Lett. 23, 13572–13580.

[ref65] Price M. N. Dehal P. S. Arkin A. P. (2010). FastTree 2—approximately maximum-likelihood trees for large alignments. PLoS One 5:e9490. doi: 10.1371/journal.pone.0009490, 20224823 PMC2835736

[ref9004] Redgrave L. S. Sutton S. B. Webber M. A. Piddock L. J. V. (2014). Fluoroquinolone resistance: mechanisms, impact on bacteria, and role in evolutionary success. Trends Microbiol. 22, 438–445. doi: 10.1016/j.tim.2014.04.007, 24842194

[ref66] Riley L. W. (2014). Pandemic lineages of extraintestinal pathogenic *Escherichia coli*. Clin. Microbiol. Infect. 20, 380–390. doi: 10.1111/1469-0691.12646, 24766445

[ref67] Robertson J. Nash J. H. E. (2018). MOB-suite: software tools for clustering, reconstruction and typing of plasmids from draft assemblies. Microb. Genom. 4:e000206. doi: 10.1099/mgen.0.000206, 30052170 PMC6159552

[ref68] Roer L. Johannesen T. B. Hansen F. Stegger M. Tchesnokova V. Sokurenko E. . (2018). CHTyper, a web tool for subtyping of extraintestinal pathogenic *Escherichia coli* based on the *fumC* and *fimH* alleles. J. Clin. Microbiol. 56, e00063–e00018. doi: 10.1128/JCM.00063-18, 29436420 PMC5869820

[ref69] Sanchez G. V. Babiker A. Master R. N. Luu T. Mathur A. Bordon J. (2016). Antibiotic resistance among urinary isolates from female outpatients in the United States in 2003 and 2012. Antimicrob. Agents Chemother. 60, 2680–2683. doi: 10.1128/AAC.02897-15, 26883714 PMC4862481

[ref70] Seemann T. (2015) Snippy: fast bacterial variant calling from NGS reads. Available online at: https://github.com/tseemann/snippy (Accessed July 1, 2026)

[ref71] Seemann T. (2016) ABRicate: mass screening of contigs for antiobiotic resistance genes. Available online at: https://github.com/tseemann/abricate (Accessed June 30, 2026)

[ref72] Silvester N. Alako B. Amid C. Cerdeño-Tarrága A. Clarke L. Cleland I. . (2018). The European nucleotide archive in 2017. Nucleic Acids Res. 46, D36–D40. doi: 10.1093/nar/gkx1125, 29140475 PMC5753375

[ref73] Sora V. M. Meroni G. Martino P. A. Soggiu A. Bonizzi L. Zecconi A. (2021). Extraintestinal pathogenic *Escherichia coli*: virulence factors and antibiotic resistance. Pathogens 10:1355. doi: 10.3390/pathogens10111355, 34832511 PMC8618662

[ref74] Souvorov A. Agarwala R. Lipman D. J. (2018). SKESA: strategic k-mer extension for scrupulous assemblies. Genome Biol. 19:153. doi: 10.1186/s13059-018-1540-z, 30286803 PMC6172800

[ref75] Spurbeck R. R. Dinh P. C. Jr. Walk S. T. Stapleton A. E. Hooton T. M. Nolan L. K. . (2012). *Escherichia coli* isolates that carry *vat, fyuA, chuA*, and *yfcV* efficiently colonize the urinary tract. Infect. Immun. 80, 4115–4122. doi: 10.1128/IAI.00752-12, 22966046 PMC3497434

[ref76] Tchesnokova V. Radey M. Chattopadhyay S. Larson L. Weaver J. L. Kisiela D. . (2019). Pandemic fluoroquinolone resistant *Escherichia coli* clone ST1193 emerged via simultaneous homologous recombinations in 11 gene loci. Proc. Natl. Acad. Sci. USA 116, 14740–14748. doi: 10.1073/pnas.1903002116, 31262826 PMC6642405

[ref77] Terlizzi M. E. Gribaudo G. Maffei M. E. (2017). Uropathogenic *Escherichia coli* (UPEC) infections: virulence factors, bladder responses, antibiotic, and non-antibiotic antimicrobial strategies. Front. Microbiol. 8:1566. doi: 10.3389/fmicb.2017.01566, 28861072 PMC5559502

[ref78] Usein C. R. Condei M. Cristea D. Ciontea S. A. Tatu-Chiţoiu D. Cristea V. . (2014). *Escherichia coli* ST131 causing invasive infections in Romanian patients—a threat we can no longer ignoreRoum. Arch. Microbiol. Immunol 73, 5–8. 25518564

[ref79] Usein C. R. Grigore L. A. Georgescu R. M. Băltoiu M. C. Condei M. Teleman M. D. (2011). Phylogenetic background and extraintestinal virulence genotypes of *Escherichia coli* vaginal strains isolated from adult women. Rev. Rom. Med. Lab. 19, 35–45.

[ref80] Waters N. R. Abram F. Brennan F. Holmes A. Pritchard L. (2020). Easy phylotyping of *Escherichia coli* via the EzClermont web app and command-line tool. Access Microbiol. 2:acmi000143. doi: 10.1099/acmi.0.000143, 33195978 PMC7656184

[ref81] WHO Regional Office for Europe, and European Centre for Disease Prevention and Control (2025). Surveillance of Antimicrobial Resistance in Europe, 2024 data: Executive Summary. Copenhagen: WHO Regional Office for Europe.

[ref82] Wirth T. Falush D. Lan R. Colles F. Mensa P. Wieler L. H. . (2006). Sex and virulence in *Escherichia coli*: an evolutionary perspective. Mol. Microbiol. 60, 1136–1151. doi: 10.1111/j.1365-2958.2006.05172.x, 16689791 PMC1557465

[ref83] Yamaji R. Rubin J. Thys E. Friedman C. R. Riley L. W. (2018). Persistent pandemic lineages of uropathogenic *Escherichia coli* in a college community from 1999 to 2017. J. Clin. Microbiol. 56:e01834-17. doi: 10.1128/JCM.01834-17 29436416 PMC5869836

[ref84] Zeng Q. Wang S. Xiao S. Liu H. Gu F. Han L. . (2025). Comparative genomic and phenotypic analysis of *Escherichia coli* ST1193 and ST131 from urinary and bloodstream infections: insights into resistance, virulence, and divergent strategies. BMC Infect. Dis. 25:1732. doi: 10.1186/s12879-025-12044-5, 41444535 PMC12729012

[ref85] Zhang J. Wen S. Duan B. Jing X. Yang K. Zhai X. . (2026). Genomic insights into selected multidrug-resistant and ESBL-producing uropathogenic *Escherichia coli* isolates in central inner Mongolia. Front. Microbiol. 17:1844989. doi: 10.3389/fmicb.2026.1844989, 42293524 PMC13260427

[ref86] Zhu L. C. Lu J. W. Wang J. Xu T. Xu J. H. (2018). Analyses on distribution and structure of *bla*_CARB-2_ in *Klebsiella pneumoniae*. Yi Chuan 40, 593–600. doi: 10.16288/j.yczz.18-034, 30021722

